# Reusable kaolin impregnated aminated chitosan composite beads for efficient removal of Congo red dye: isotherms, kinetics and thermodynamics studies

**DOI:** 10.1038/s41598-022-17305-w

**Published:** 2022-07-28

**Authors:** Mohamed M. Abou Alsoaud, Mahmoud A. Taher, Abdelrahman M. Hamed, Mohamed S. Elnouby, Ahmed M. Omer

**Affiliations:** 1 Department of Chemistry, Faculty of Science, AL-Azhar University (Assuit Branch), Cairo, Egypt; 2 Nanomaterials and Composites Research Department, Advanced Technology and New Materials Research Institute (ATNMRI), City of Scientific Research and Technological Applications (SRTA-City), New Borg El-Arab City, P.O. Box: 21934, Alexandria, Egypt; 3 Polymer Materials Research Department, Advanced Technology and New Materials Research Institute (ATNMRI), City of Scientific Research and Technological Applications (SRTA-City), New Borg El-Arab City, P. O. Box: 21934, Alexandria, Egypt

**Keywords:** Nanocomposites, Pollution remediation, Polymer characterization

## Abstract

In this investigation, Kaolin (K) impregnated aminated chitosan (AM-CTS) composite beads were fabricated with multi-features including low-cost, high performance, renewable and ease of separation for adsorption of anionic Congo red (CR) dye. Characterization tools such as FTIR, XRD, SEM, TGA, BET, XPS and Zeta potential were thoroughly employed to confirm the successful formulation process. The results revealed that K@ AM-CTS composite beads displayed higher specific surface area (128.52 m^2^/g), while the thermal stability was prominently improved compared to pure AM-CTS. In addition, the adsorption equilibrium of CR dye was accomplished rapidly and closely gotten within 45 min. The removal efficiency was significantly enriched and reached 90.7% with increasing kaolin content up to 0.75%, compared to 20.3 and 58% for pristine kaolin and AM-CTS, respectively. Moreover, the adsorption process obeyed the pseudo-first order kinetic model, while data were agreed with the Freundlich isotherm model with a maximum adsorption capacity reached 104 mg/g at pH 6. Furthermore, D–R isotherm model demonstrated the physical adsorption process of CR dye, which includes the electrostatic interactions, ion exchange and H-bonding. Thermodynamics evidenced the spontaneous and endothermic nature of the adsorption process. Interestingly, the developed K@AM-CTS composites beads showed better reusability for eight consecutive cycles, suggesting their feasible applicability for adsorptive removal anionic dyes from polluted aquatic bodies.

## Introduction

Massive quantities of dyes are being applied in many industrial fields like pigments, textiles, papers, plastics, printing, cosmetics and food coloring^1^. Discharging of these dyes has harmful impacts on human health and aquatic life owing to their chemical stability and non-biodegradability^2^. Congo red (CR) or 1-naphthalenesulfonic acid, 3,3′-(4,4′-biphenylenebis (azo)) bis (4-amino-) disodium salt is a heterocyclic aromatic benzidine-based anionic diazo dye^3^. As direct dyes family, CR is simply soluble in water^4^, which facilitate their direct application in textile industry for dying cellulosic fibers^5^. Furthermore, CR is a well-known histological dye, which used effectively to prove the presence of amyloidal deposits in tissue, in addition to its use for dying the cells walls of fungi and plants, Gram-negative bacteria^6^. Nevertheless, CR dye has a carcinogenic effects, allergic dermatitis and skin irritation^5^. Therefore, it is urgent to find efficient materials for removing of CR dye from water bodies. Several plentiful water remediation techniques have been employed for removing noxious dyes such as photocatalytic degradation^7^, coagulation^8^, membrane separation^9^, ion exchange^10^, ozonation^11^ and adsorption^12^. Primarily, there are strict criteria to choose the appropriate dye removal technique such as simple design/operation, energy-saving and economical cost-effectiv ^13^. Adsorption technique has been chosen as the most technique adopted for adsorption of dyes owing to its beneficial features such as high performance, minimal secondary pollutants and simple processing^3,5^. Accordingly, the growth of eco-friendly and effectual adsorbents with the supreme aptitude for adsorption has become a great demand for researchers.

Numerous adsorbent materials such as clays^14^, carbon based-materials^15^ and polymers^16^ are the most common materials applied as adsorbents. Recently, adsorbents based-natural polymers have gained much interest owing to their effectual adsorption performance toward various pollutants\from industrial water bodies^17^. Among them, chitosan (CTS) is a type of a natural polycationic carbohydrate biopolymer, created from the deacetylation of chitin biopolymer that considered the main component of the exoskeleton of crustacean shells^18^. CTS composed of un-branched chains of B-(1-4)-2-acetoamido-2-deoxyd-glucose^19^. CTS demonstrates distinctive properties such as hydrophilicity, eco-friendly, biocompatibility, biodegradability, ease of modification and lack of toxicity^20^. Accordingly, it has been extensively employed in various applications including biomedical, industrial, food packaging and water treatment fields^21,22^. Due to its reactive amino and hydroxyl groups^23^, CTS has been effectively applied as efficient adsorbent for removing of various contaminants from their aquatic systems such as toxic organic dyes^24^, heavy metals^25^, pharmaceutical residues^26^ and oil spills^27^. Nevertheless, pure CTS suffer from its low adsorption kinetics, limited surface area and low mechanical characteristics. To overcome these drawbacks, several modification processes such as grafting^28^, carboxymethylation^29^, sulfonation^30^, amination^31^, Schiff base^32^ and composite formation^33^ have been employed to pristine CTS.

Chitosan-based composites have attracted great attention in various applications due to their impressive characteristic such as mechanical strength, chemical stability, surface area and structural properties^34^**.** A plethora of the materials such as carbon-based materials, clay, and metal/mixed oxide nanoparticles have been used for the removal of toxic dyes from their aquatic media^35^. Incorporation of various clays into CTS matrix is a viable solution for providing superior adsorption efficiency, higher stability, extraordinary surface area and special catalytic activity^36^. Among them, Kaolin (K) is a hydrated aluminum silicate, which considered one of the most common natural inorganic clays. Kaolin is an indispensable material for various industrial processes owing its excellent properties such as abundant availability, environmental friendly, low-cost production, high surface area, good bonding ability, high whiteness, low-cost and excellent thermal stability^37^. Though, aggregation and the limited adsorption capability of kaolin towards the anionic species are considered the most common disadvantages that limit its application as an adsorbent material on a large-scale^38^. Thus, several reports have focused on the modification of Kaolin to overcome these weaknesses including surface modification and composite formation^39^.

The present study deals with the development of eco-friendly and low-cost chitosan-based composites. Up to now, no studies involved the fabrication of AM-CTS/Kaolin composite beads for adsorptive removal of noxious anionic dyes. Hence, an attempt was made to fabricate new multi-featured adsorbent beads based on a newly developed aminated chitosan (AM-CTS) and Kaolin. Herein, we aimed to combine the individual features of chitosan derivative and Kaolin and to overcome their individual drawbacks via the formation of easy-separable composite beads. The extra amine groups in AM-CTS derivative are expected to enrich the adsorption characteristics toward anionic dyes compared to the pristine chitosan. In addition, impregnation of Kaolin into AM-CTS beads would overcome the limited surface area of AM-CTS derivative and enhances its adsorption capacity and recyclability. Furthermore, the developed composite beads would provide facile separation of adsorbent from adsorption media without centrifugation and filtration systems. The developed K@ AM-CTS composite beads were well-characterized their structures, thermal and morphological properties using several characterization tools. The ability of the developed adsorbent to adsorb anionic CR dye under various adsorption conditions was examined. Furthermore, kinetics, isotherms and thermodynamics were thoroughly studied. Besides, the ability of K@ AM-CTS to reuse for several consecutive cycles was also evaluated.

## Experimental

### Materials

Chitin (degree of acetylation = 0.94) was delivered from Daejung (Korea). *Para*-benzoquinone (PBQ; 99%), Ethylene-diamine (EDA; 99%) and Kaolin were supplied from the Huaiyuan Mining Industry Co Ltd. (China). Ammonium hydroxide (99%), Sodium hydroxide (98%), Hydrochloric acid (37%) and Acetic acid (98%) were purchased from Loba Chemie (India). Congo red dye was bought from Sigma-Aldrich Co. (Germany), while its characteristics were deliberated in Supplementary Table S1.

### Synthesis of aminated chitosan derivative (AM-CTS)

(AM-CTS) was prepared according to the authors previous work^31^. In brief, the OH groups of chitin were firstly activated via immersing of chitin (10 g) into PBQ solution (6.9 mM; pH 10) under stirring. The activation process was conducted at 60 °C for 6 h. Thereafter, PBQ-activated chitin was washed by distilled H_2_O to remove the excess PBQ and followed by soaking in EDA solution (6.9 mM) for 6 h at 60 °C under constant stirring. The obtained aminated chitin was separated and washed several times using distilled H_2_O to remove the unreacted EDA molecules. Later, aminated chitin was deacetylated by NaOH (50%) at 100 °C for 18 h under stirring. The resultant aminated chitosan (AM-CTS) was filtrated, washed with distilled H_2_O and dried at 50 °C.

### Formulation of K@AM-CTS composite beads

**Figure 1:**
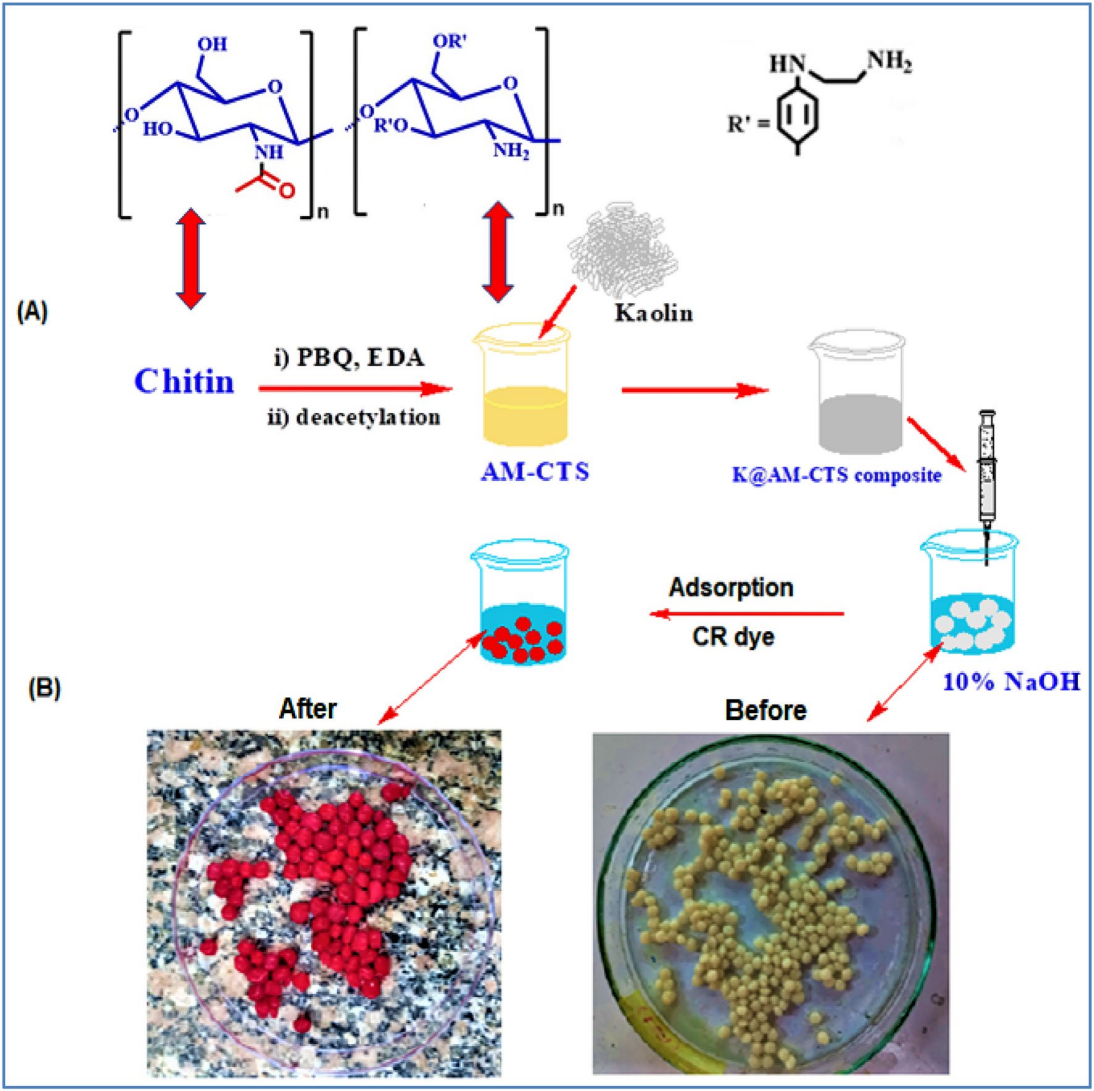
(**A**) A schematic representation for the formulation of K@AM-CTS composite beads and (**B**) digital laboratory images of freshly prepared beads before and after adsorption of Congo red dye.

AM-CTS solution was prepared by dissolving it in 1% (v/v) of glacial acetic acid solution at room temperature under stirring to have final concentration of 3% (w/v). Next, Kaolin (0.3, 0.5, 0.75 and 1%) was dispersed in 5 mL distillated water under ultra-sonication for 30 min, then added drop-by-drop to AM-CTS solution. The mixture was left for 2 h under continuous stirring (250 rpm) at room temperature to have homogenous mixture. Finally, the composite was dropped under moderate stirring into NaOH solution (10%; w/v) using a plastic syringe (5 cm^3^). After 30 min, the formulated K@AM-CTS composite beads were gently separated, washed multiple times with distilled water and dried at 50 °C. Figure 1 illustrates the preparation of K@ AM-CTS composite beads and laboratory images for the developed composite beads.

### Characterization

Fourier Transform Infrared Spectroscopy (FTIR; Shimadzu—8400 S, Japan) was used to investigate the chemical structure and functional groups of the developed composite beads. The thermal properties were checked by the thermal gravimetric analyzer (TGA; Shimadzu-50, Japan). Scanning Electron Microscope (SEM; Joel Jsm 6360LA- Japan) was used to examine the morphological changes of composites beads. X-ray diffraction (XRD) was employed to inspect the crystallinity of composite beads. X-ray photoelectron spectroscope (XPS, Axis Ultra DLD, Shimadzu, Japan) was employed for investigating the elemental-surface composition. Besides, BET (Brunauer, Emmett and Teller) and Zeta potential (Malvern, UK) were used to determine the surface area and the surface charges of the composite beads.

### Batch adsorption studies

The developed composite beads (0.05–0.3 g) were added to 25 mL of CR dye solution (25–200 mg/L), while the pH medium was adjusted in the range of 4–10 using 0.1 M of both NaOH and HCl solutions. The temperature of the adsorption medium was examined in the range of 25–55 °C, while the under stirring speed was varied from 50 to 250 rpm. After time intervals (1–180 min), the residual CR dye concentration was estimated at 497 nm using a UV–Vis spectrophotometer. The adsorption capacity at equilibrium q_e_ (mg/g) and removal (%) were measured according to the following equations^40^:

1 $$\mathrm{q}{\text{e}} =\frac{(\mathrm{Co}-\mathrm{Ce})\mathrm{V}}{\mathrm{W}},$$

2 $$\mathrm{q}{\text{t}}=\frac{(\mathrm{Co}-\mathrm{Ct})\mathrm{V}}{\mathrm{W}},$$

3 $$\mathrm{R }\left(\mathrm{\%}\right)=\frac{\left(\mathrm{Co}-\mathrm{Ct}\right)}{\mathrm{Co}} \times 100,$$

where, q_e_ and q_t_ (mg/g) are adsorption capacity at equilibrium and time t, respectively. C_o_ and Ct (mg/L) are the CR concentration at 0 and t time, respectively. V is the volume of CR (L) and W is the weight of dried adsorbent (g).

### Reusability test

Reusability test was performed to assess the ability of the K@AM-CTS composite beads to reuse for adsorption of CR dye. In brief, the developed adsorbent beads were collected after completion the adsorption process, followed by dipping at room temperature in 25 mL of the Methanol/NaCl solution mixture as a desorption medium under continuous stirring for 1 h. The regenerated beads were separated and subjected for several adsorption–desorption cycles.

## Results and discussion

### Characterization of adsorbent

#### FTIR

**Figure 2:**
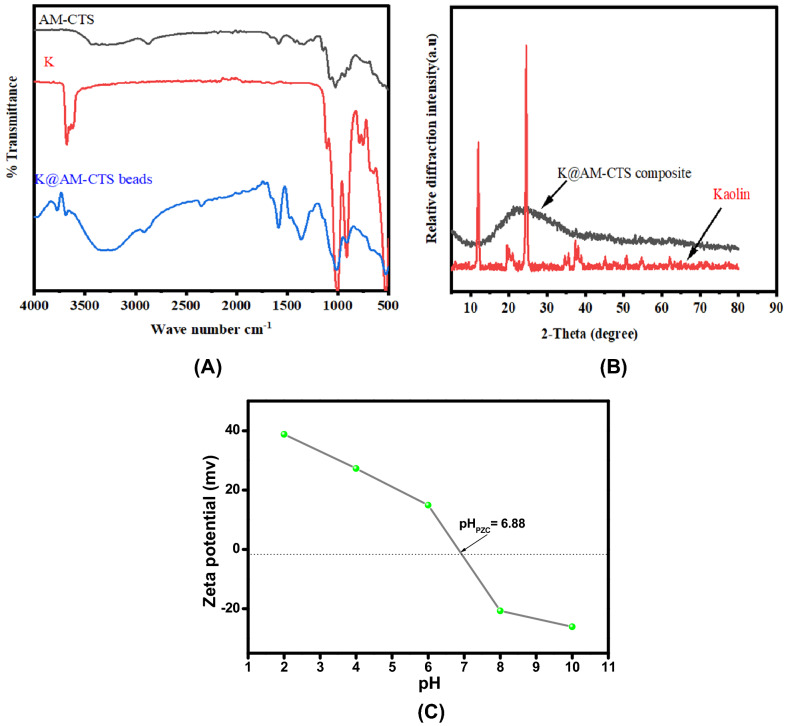
(**A**) FTIR of AM-CTS, kaolin and K@AM-CTS composite beads, (**B**) XRD patterns of kaolin and K@AM-CTS composite beads and (**C**) Zeta potential profile of K@AM-CTS composite beads.

The infrared spectra of AM-CTS, kaolin and K@AM-CTS composite beads are shown in Fig. 2A. The FTIR spectrum of AM-CTS shows the main characteristic peaks of polysaccharides^41^. The absorption broad at 3279 cm^−1^ is attributed to stretching vibration of overlapped –OH and NH_2_ functional groups. The observed broad bands at 2873, 2186, 1023 and 1583 cm^−1^ are correspond to CH_2_, C–OH stretching, C–N groups and N–H bending vibrations, respectively. Also, there are two bands at 1335 and 2873 cm^−1^ could be ascribed to in-plane bending and stretching vibration of C–H group, respectively. On the other hand, the IR spectrum of kaolin displays an absorption band at around 3680 cm^−1^ which is attributed to the –OH stretching vibrations of water molecules on the external layer of kaolin in addition to the Al_2_OH groups of the octahedral layer. The typical peak at 1108 cm^−1^ could be related to the stretching vibration of Si–O–Si and O–Si–O of kaolin. The band around 1001 cm^−1^ could be assigned to the stretching vibration of the Si–O groups. Furthermore, the distinctive peaks at 526–647 cm^−1^ are attributed to Al–O–Si and Si–O–Si bending vibrations^42^. Besides, FTIR spectrum of K@AM-CTS composites beads explains the essential peaks of the original materials comparing the IR spectrum of pristine AM-CTS and Kaolin suggesting that characteristic bands of both AM-CTS and Kaolin are absolutely present in the composite. The detected shift in N–H bending deformation band from 1583 cm^−1^ in pure AM-CTS to 1584.86 cm^−1^ in K@AM-CTS, in addition to the noticed shift of stretching vibration of –OH and NH_2_ from lower wavelength (3279 cm^−1^) to the higher one (3312 cm^−1^). Furthermore, the C–H band at 2873 cm^−1^ was moved also to a higher wavelength of 2915 cm^−1^, indicated the interaction of the negatively charged sites of the kaolin with the protonated amine groups (−NH_3_^+^) of AM-CTS^43^, confirming the successful formation of K@AM-CTS composite.

#### XRD

Figure 2B illustrates the XRD patterns of natural kaolin and K@AM-CTS composite beads. The main peaks of pure kaolin found at 2θ = 12.34°, 20.36°, 24.88°, 35.94° and 37.76°, these results are in good agreement with that reported elsewhere^44^. In addition, the main crystal size was 24.67 nm at maximum intensity peak around 24.88° which was calculated according to the reported Debye Scherer’s equation^31^. The XRD pattern of K@AM-CTS composite beads shows more amorphous in nature. Where the crystal structure of kaolin disappeared and not noticeable by addition of kaolin to AM-CTS, in addition the distinct broad peaks around 2θ = 15°–35° were appeared in K@AM-CTS composite beads compared to pure of kaolin, which may be indicating AM-CTS entered into the interlayer spacing of kaolin and created process was achieved successfully.

#### Zeta potential

The determination of point of zero charge (PZC) was achieved to investigate the surface charge and acidic-basic character of the developed adsorbent beads^37^. ZP measurements (Fig. 2C) elucidated that PZC value of K@AM-CTS composite beads was 6.88. This finding suggested that the surface of K@AM-CTS was positively charged at pH < 6.88, which is expected to generate columbic interactions with the negatively charged CR dye. Conversely, at pH > 6.88, the surface of the beads was negatively charged. In the light of the above mentioned results, K@AM-CTS composite beads are suitable to adsorb both cationic and anionic pollutants via the electrostatic interactions, endowing our fabricated composite beads one more merit.

#### BET

**Figure 3:**
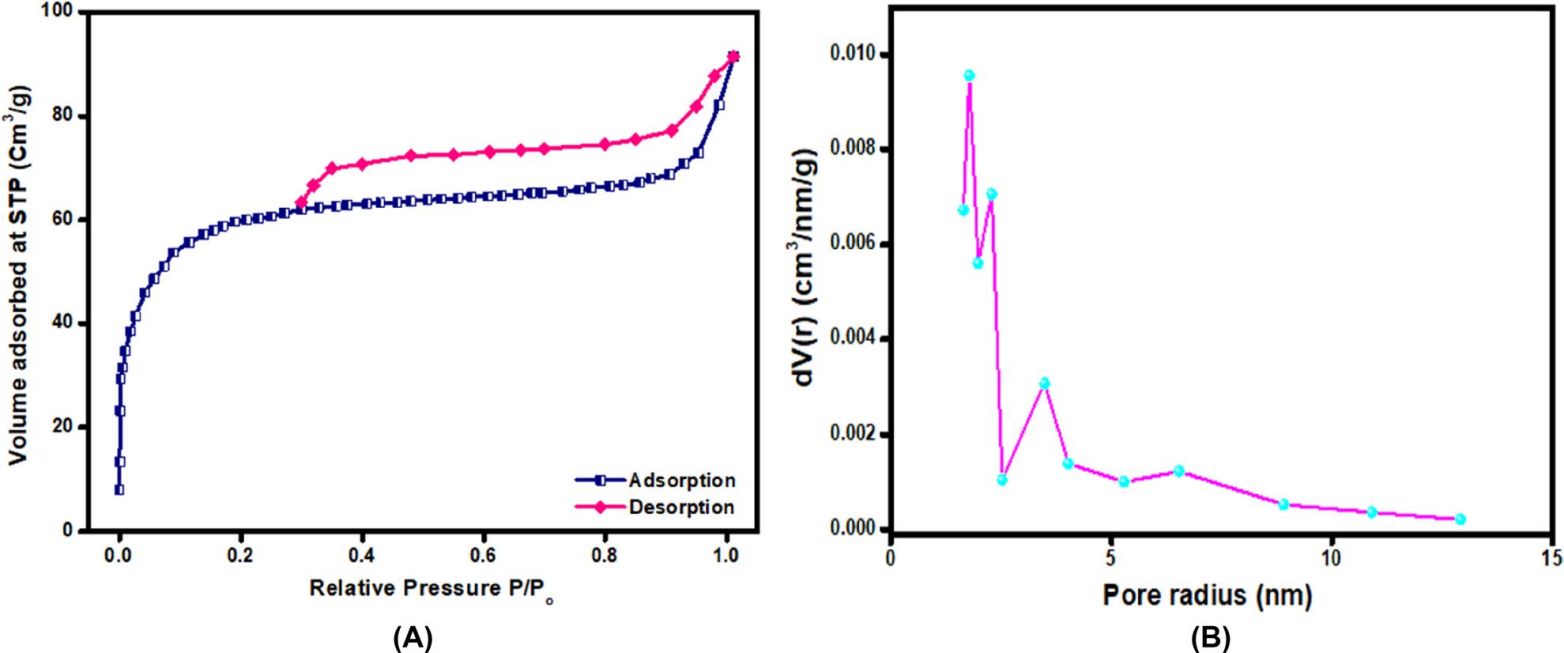
(**A**) N2 adsorption/desorption isotherm and (**B**) pore size distribution of K@AM-CTS composite beads.

The N_2_ adsorption/desorption isotherm and the pore size distribution of K@AM-CTS composite were investigated as shown in Fig. 3A,B. The BET isotherm points out the mesoporous structure of K@AM-CTS composite beads, since the hysteresis loop represents type IV with H4. Furthermore, the S_BET_ of K@ AM-CTS was found to be 128.52 m^2^/g with average pore size 2.056 nm^45^.

#### TGA

The thermal properties of the fabricated K@AM-CTS composites beads were studied using TGA analysis at the temperature range from 25 to 800 °C, while the gained data were summarized in Table 1. The results referred that both AM-CTS and K@AM-CTS composite beads demonstrated three stages of weight loss. The first stage was detected at the ambient temperature (up to 120 °C) and recorded maximal weight loss of 24.2 and 8.5% for AM-CTS and K@AM-CTS composite beads, respectively. The first degradation stage was attributed to the evaporation of residual moisture from examined samples^46^. The second stage was achieved with rising temperature up to 400 °C, which recorded 54.8 and 44.7% of weight loss for AM-CTS and K@AM-CTS composite beads, respectively. This degradation stage could be assigned to decomposition of saccharide rings. The results signified also that the composite beads displayed better thermal stability compared to pristine AM-CTS due to the existence of kaolin. The third degradation stage concerned with the complete decomposition of matrix which was observed with further elevating temperature up to 800 °C. Besides, the temperature required for composite beads to loss its half weight (T_50%_ °C) was 443 °C compared to 370 °C for native AM-CTS, proving the adequate thermal stability of the fabricated composite beads^31^.

**Table 1:** TGA data of AM-CTS and K@AM-CTS composite beads.

Sample	(%) Weight loss at Ambient 0–120 °C	(%) Weight loss at 400 °C	T_50%_ °C
AM-CTS	24.2	54.8	370
K@AM-CTS composite beads	8.5	44.7	443

#### Morphological properties

**Figure 4:**
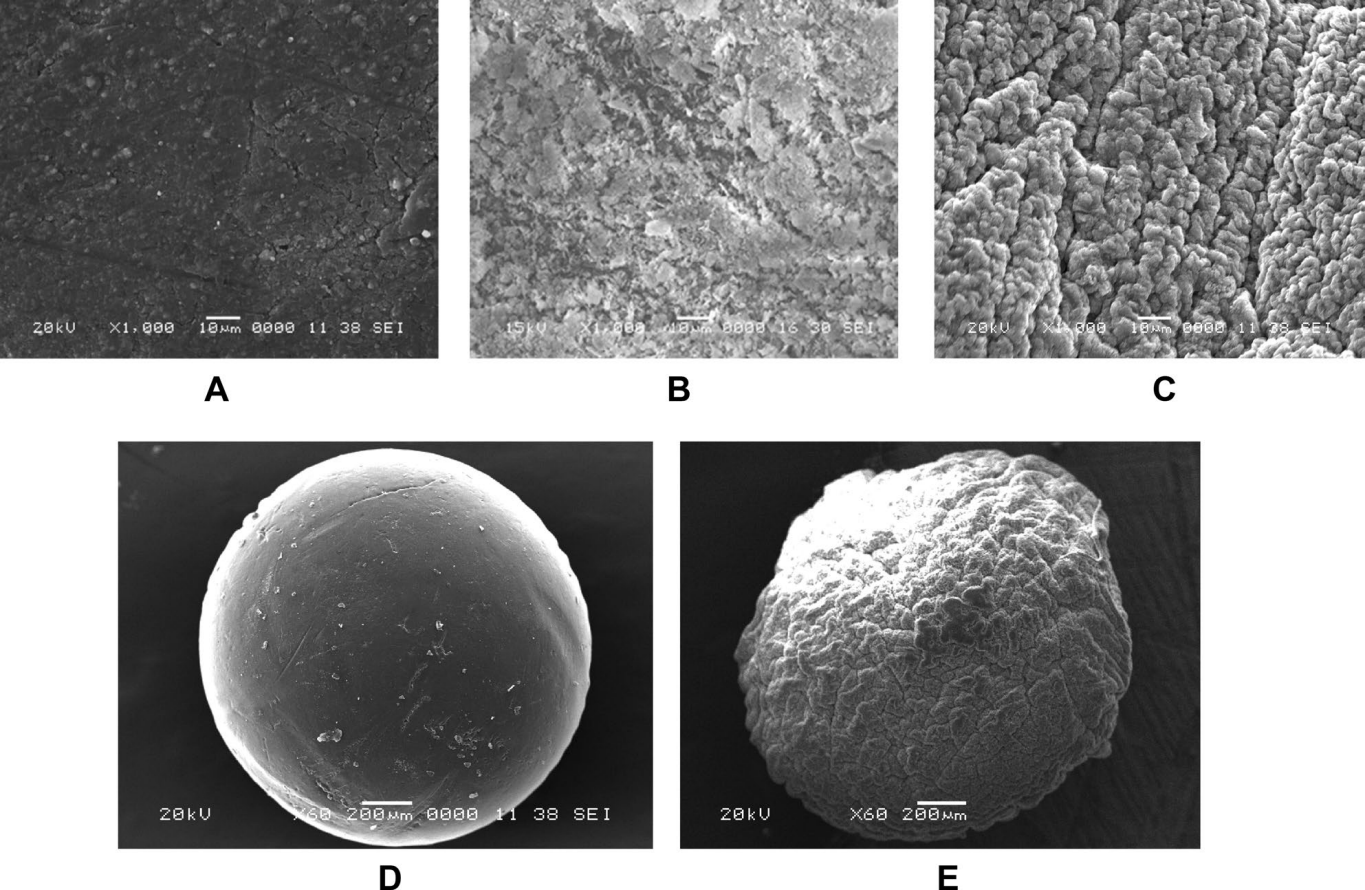
SEM images of (**A**,**D**) pure AM-CST beads, (**B**) kaolin and (**C**,**E**) K@AM-CTS composite beads at different magnifications.

SEM images of the pristine AM-CTS, kaolin and the formulated K@AM-CTS composite beads were deliberated in Fig. 4. It was clear that AM-CTS (Fig. 4A) display a rough surface with some granules with different sizes. In addition, the surface of kaolin (Fig. 4B) showed nano chips accumulated on top of each another which are represent the solid hexagonal in Kaolin shape^47^. On the other hand the SEM image of the composite beads K@AM-CTS composites beads (Fig. 4C) demonstrated rougher and crusty surface with more crinkles, as a result of accumulation of kaolin onto AM-CTS surface^48^. These changes were confirmed by SEM images of the whole beads. It was observed that the whole surface of AM-CTS beads (Fig. 4D) showed a spherical form with a few particles and unified pores, while the whole surface was entirely changed after the formation of composite beads to non-spherical, wrinkled and rougher surface (Fig. 4E). The observed morphological changes confirm the successful formation of the composite beads.

#### XPS analysis

**Figure 5:**
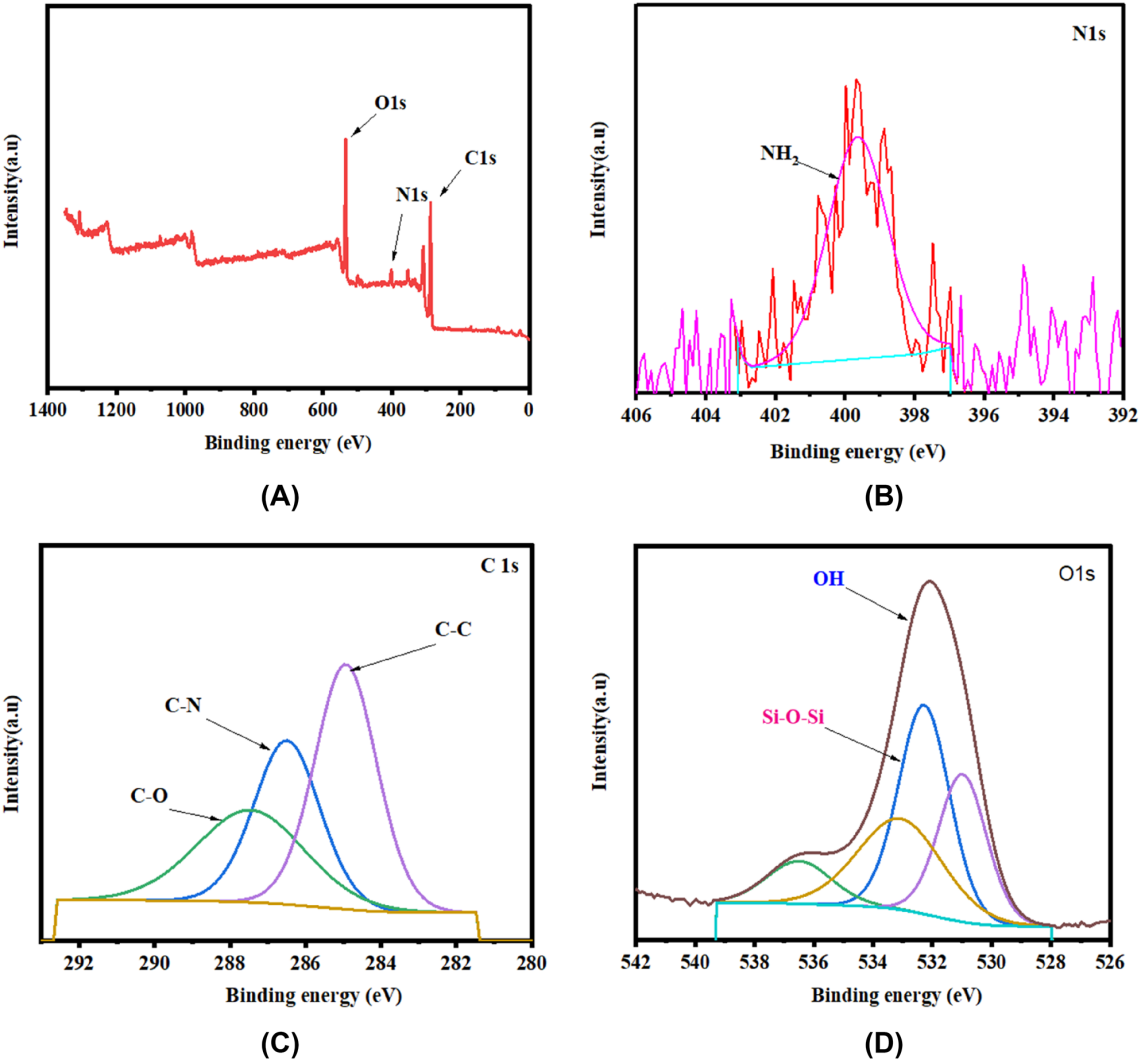
XPS spectra for (**A**) wide scan of K@AM-CTS composite beads, (**B**) N1s, (**C**) C1s and (**D**) O1s.

The XPS analysis was performed to investigate the main elements in K@AM-CTS composite beads before and after adsorption, in addition to clarify the reactions among composite beads and CR dye^49^. The results demonstrated that the composite beads before adsorption of CR dye mostly contain the following elements: C1s, O1s, and N1s at binding energy (BE) of 287.33, 534.36 and 401.91 eV respectively, as demonstrated in a wide-scan spectrum XPS (Fig. 5A). In addition, the high-resolution of N1s (Fig. 5B) exhibited the unique peak of NH_2_ group at BE of 399.7 eV. Furthermore, the high-resolution of C1s (Fig. 5C) showed three peaks at BE of peaks at 284.93, 286.43 and 287.02 eV, which were attributed to C–C, C–N and C–O, respectively^50^. Besides, the high-resolution of O s (Fig. 5D) illustrated the two peaks at BE of 532.25 and 533.6 eV due to the presence of OH groups and Si–O–Si of kaolin clay.

### Factors affecting the adsorption of CR dye

#### Impact of Kaolin ratio

**Figure 6:**
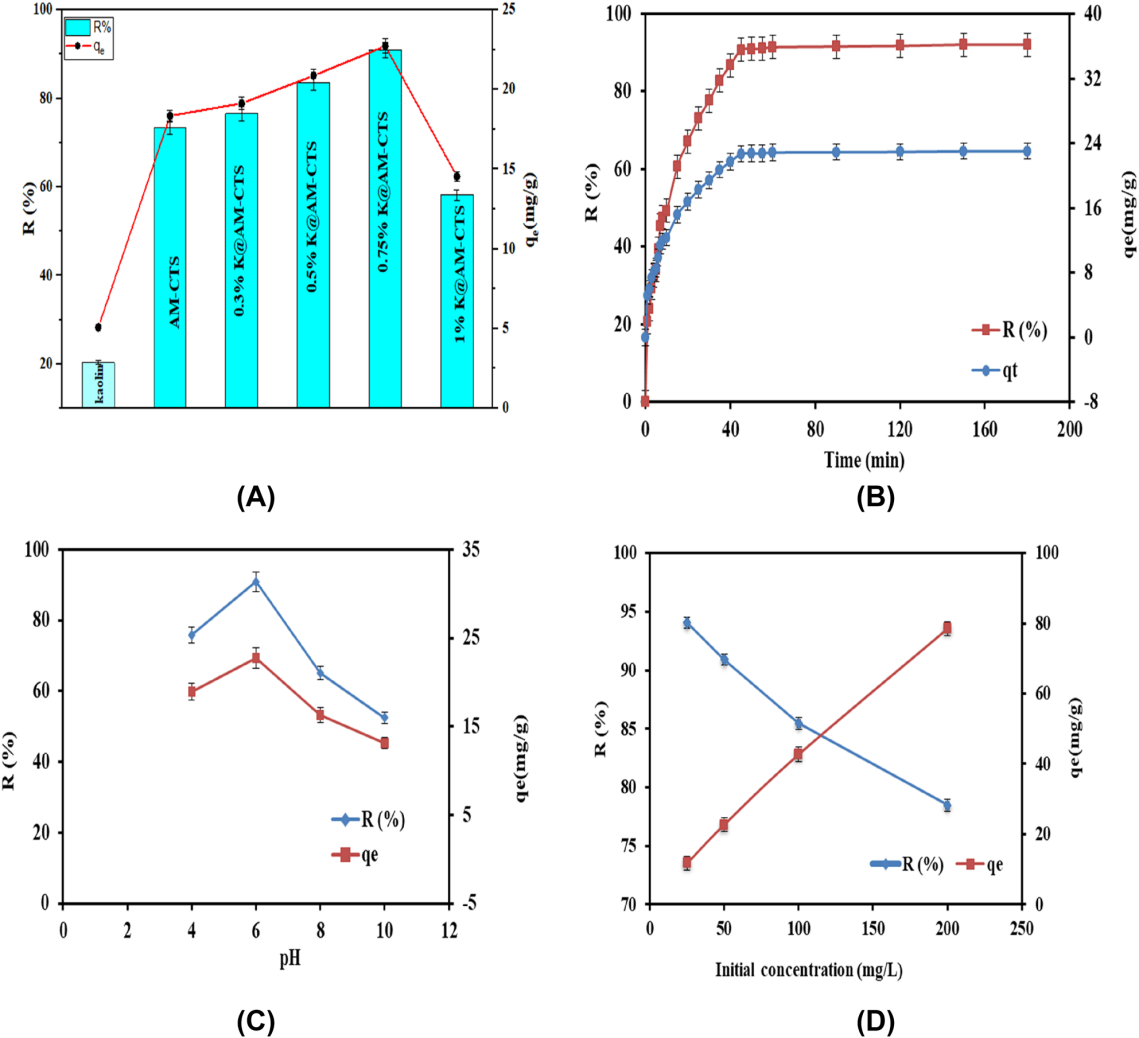
Impacts of (**A**) Kaolin ratio, (**B**) contact time, (**C**) pH and (**D**) initial concentration of CR dye.

As depicted in Fig. 6A, incorporation of kaolin clay up to 0.75% significantly enhanced the removal efficiency of the composite beads. The removal (%) increased respectively in the order of 0.75% K@AM-CTS (90.7%) > 0.5% K@AM-CTS (83.5%) > 0.3% K@AM-CTS (76.5%) > AM-CTS (73.3%) > 1% K@AM-CTS (58%) > kaolin (20.33%). Similarly, the adsorption capacity was increased from 18 to 22.7 mg/g with increasing kaolin content from 0.3 to 0.75%. On the other hand, the removal (%) of CR dye was significantly decreased from 90 to 58% with further increasing kaolin content up to 1%. These results could be ascribed to blocking of AM-CTS pores with further rising Kaolin content, in addition to the limited affinity of Kaolin towards anionic species^39^.

#### Impact of contact time

The study of adsorption contact time is significant for determining the equilibrium adsorption time of CR by K@AM-CTS composite beads. As illustrated in Fig. 6B, the results signified that the removal (%) and adsorption capacity were significantly boosted, since it recorded about 20% and 5.2 mg/g within the first minute. In addition, the rate of removal increased rapidly up to 50% with increasing time up to 10 min as a result of the large number of the active adsorption sites on K@AM-CTS composite beads. Moreover, the removal (%) increased gradually from 50 to 91% with further increasing contact time up to 45 min. Although, there is no noticeable increase in the removal efficiency and adsorption capacity with increasing time beyond 45 min, since most of the active site of K@AM-CTS composite beads were occupied by CR dye molecules and reached the equilibrium within 45 min.

#### Impact of pH

The pH value of the creative solution is a crucial factor to determine the best removal efficiency for CR dye. Herein, the impact of pH on the adsorption profiles was studied in the range of 4–10. Figure 6C clarified that the removal (%) and adsorption capacity were increased gradually from 75.8% and 18.9 mg/g to 91% and 22.7 mg/g, respectively with rising pH value from 4 to 6. These results could be explained by increasing the electrostatic interactions between sulfonic acid group (SO_3_^−^) of CR and extra positively charged NH_2_ groups on the surface of K@AM-CTS composite beads^4^. On the other hand, further increasing pH value beyond 6–10 clearly decreased the removal (%) from 91 to 52%, which agreed with the obtained ZP results, since the K@AM-CTS surface carried negative charges at pH > 6.88. This decline could be attributed to deprotonation of functional groups of K@AM-CTS composite beads, resulting in a strong electrostatic repulsion between the anionic CR and the negatively charged composite beads. These results were in line with other reported studies that that evinced the change in the surface charge of CR according to the pH medium^51^. Furthermore, the present functional groups of CR dye at basic media could be protonated to the cationic form. Thence, the electrostatic repulsion forces were occurred between the positively charged K@AM-CTS and the protonated CR, causing a decrease in the removal (%) and adsorption capacity values. Accordingly, pH 6 was represented as an optimum pH value for the adsorption of CR dye, which agreed with other reported studies^4^.

#### Impact of initial concentration of CR

Commonly, the adsorption capacity and removal (%) of dye depend on the initial concentration of dye. As shown in Fig. 6D, the adsorption capacity was increased from 11.7 to 78.5 mg/g with increasing the initial concentration of CR dye from 25 to 200 mg/L. These observations could be associated with increasing the dynamic forces that overwhelms the mass transfer resistance of CR dye molecule from bulk to the K@AM-CTS composite beads surface^52^. Therefore, a greater amount of CR dyes molecules that could be adsorbed on the adsorbent surface at higher concentrations. Conversely**,** the removal (%) of CR dye deceased from 94.04 to 78.47% as a result of existence of large number of active sites that ready to adsorb more CR dye molecules at low concentrations. At higher CR concentrations, these active sites become saturated and their tendency to adsorb more dye molecules is limited.

#### Impact of adsorbent dosage

**Figure 7:**
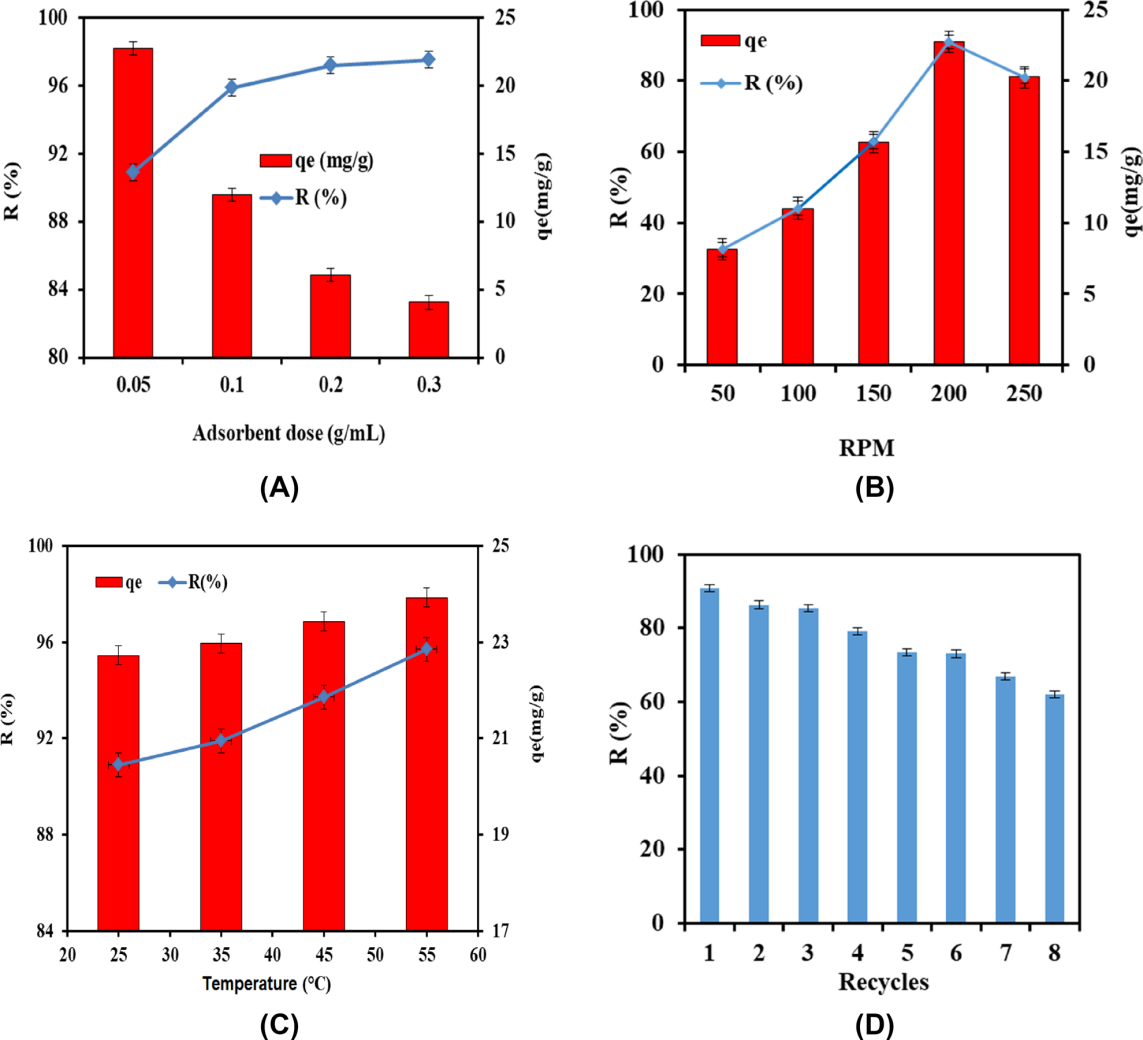
Impacts of (**A**) adsorbent dose, (**B**) agitation speed and (**C**) adsorption medium temperature and (**D**) Reusability of K@AM-CTS composite beads**.**

The influence of different adsorbent dosages (0.05–0.3 g) on the adsorption of CR dye was investigated in Fig. 7A. It was observed that the removal (%) was increased up to 97% with increasing K@AM-CTS the composite beads dosage from up to 0.3 g as a result of profusion of active sites on surface of composite beads. On the other hand, the adsorption capacity was significantly decreased with raising the adsorbent dosage due to increasing the conglomeration tendency of the adsorbent particles.

#### Impact of agitation speed

The effect of variation of agitation speed on the adsorption process was examined in the range 50–250 rpm at constant all adsorption conditions as displayed in Fig. 7B. The removal (%) and adsorption capacity values were obviously boosted from 32% and 8.1 mg/g to 91% and 22.7 mg/g with increasing agitation speed from 50 to 200 rpm, respectively. These results could be ascribed to improving the diffusion rate of CR molecules towards the adsorbent beads surface with raising the agitation speed. Thus, large number of vacant adsorbent sites is offered to adsorb CR dye. Nevertheless, further increase in the agitation speed up to 250 rpm induces the desorption tendency of CR dye as a result of deformation of the stable film, and the adsorption capacity and removal (%) values decreased accordingly^27^.

#### Impact of adsorption medium temperature

Figure 7C explained the effect of adsorption medium temperature over the range 25–55 °C on both adsorption capacity and removal (%) of CR dye. The results refereed that the adsorption capacity and removal (%) were faintly boosted from 22.7 mg/g and 91% to 24.0 mg/g and 96% with increasing temperature from 25 to 55 °C, respectively. These results confirmed that the adsorption process of CR dye on to K@AM-CTS composite beads was endothermic in nature^53^. These observations could be attributed to increasing the segmental motion of K@AM-CTS composite beads, in addition to generation of large number of active sites on the composite beads surface with increasing the adsorption temperature^54^. Moreover, the diffusion rate of CR dye molecules through the external boundary layer of K@AM-CTS composite beads increases also with rising temperature up to 55 °C^55^.

### Reusability

After proving the efficiency of the developed adsorbent beads in the adsorptive removal of anionic CR dye, it is essential to infer their reusability from economic point of view. Figure 7D clarified that the developed composite beads still retain adequate adsorption properties with a maximal removal (%) exceeded 60% after eight consecutive cycles, suggesting their potential applicability as easy-separable and reusable adsorbent for CR dye with high performance.

### Adsorption isotherms

**Figure 8:**
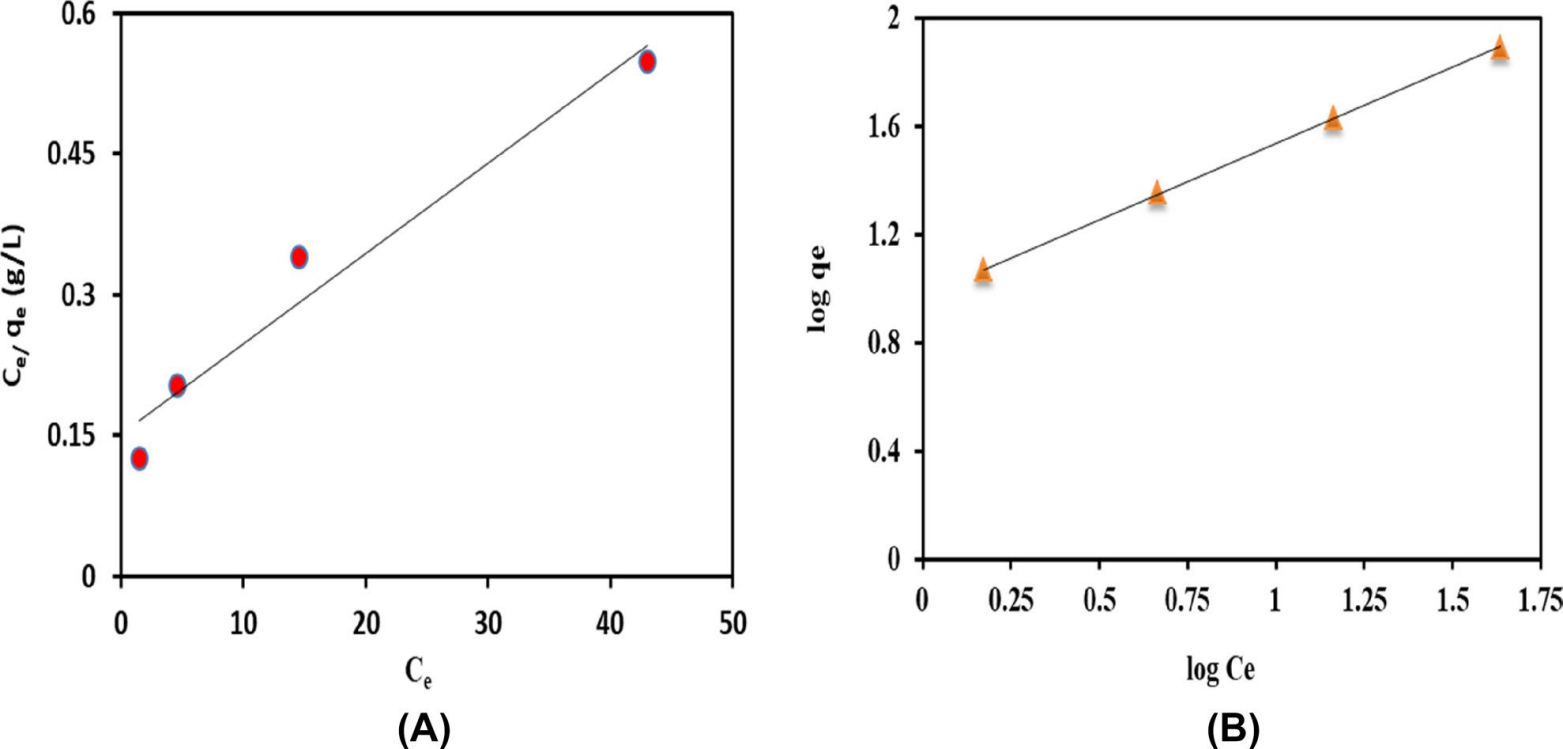
Adsorption isotherm plots of (**A**) Langmuir and (**B**) Freundlich models.

The adsorption equilibrium represented the correlation between amounts of adsorbents and different concentrations of adsorbate at specified temperature, herein, adsorption equilibrium used to describe the interaction type between K@AM-CTS composite beads and CR dye^31^. The adsorption data were explained using Freundlich, Langmuir and D–R models as presented in Table 2. The empirical Langmuir model (Fig. 8A) assumes that the adsorption process could be achieved via the formation of mono layer molecules through filling of adsorption site by one dye molecule, which is beneficial in case of a homogenous surface. In addition, this model is also useful for identify the maximum adsorption capacity of the developed composite beads. The Langmuir isotherm model can be expressed as follows^56^:

4 $$\mathrm{q}{\text{e}}=\frac{\mathrm{bq}{\text{max}}\mathrm{c}{\text{e}}}{1+\mathrm{bc}{\text{e}}},$$

5 $$\frac{\mathrm{Ce }}{\mathrm{qe}}=\frac{1}{\mathrm{bqm }}+\frac{\mathrm{Ce }}{\mathrm{qm}},$$

where, q_max_ (mg/g) is the theoretical Langmuir maximum approval (mg/g); q_e_ is the CR dye uptake at equilibrium (mg/g), C_e_ represent the equilibrium concentration of CR (mg/L) and b is the Langmuir constant (L/mg).

**Table 2:** The parameters of Freundlich, Langmuir and D–R isotherms models for adsorptive removal of CR dye.

Equilibrium model	Parameter	Value
Freundlich	n	1.77619
	k_F_ (L/mg)	9.46891
	R^2^	0.9999
Langmuir	q_m_ (mg/g)	104.16
	b (L/mg)	0.06295
	R^2^	0.9582
D–R	q_s_ (mg/g)	47
	K_ad_ (mol^2^ K^−2^ J^−2^)	9 × 10^–7^
	R^2^	0.728
	E (KJ mol^−1^)	0.745

The ultimate characteristic of the Langmuir model can be shown by dimensionless constant (R_L_) (Eq. 6), which could be used to describe the type of the adsorption process whether favorable (0 < R_L_ < 1) or unfavorable (R_L_ > 1**)** or liner (R_L_ = 1) or irreversible (R_L_ = 0)^57^.

6 $$\mathrm{R}{\text{L}}=\frac{1}{1+\mathrm{bce}},$$

where, C_e_ is the equilibrium concentration of CR (mg/L) and b is the Langmuir constant (L/mg).

Furthermore, Freundlich model (Fig. 8B) assumed that a multilayer adsorption process acquired place on a heterogeneous surface of absorbent^48^. In addition, the value of Freundlich constant (n) describes the kind of adsorption, which in case of less than 1 means that adsorption process has poor (un-favorable), while from 1–2 means that adsorption process is moderate and finally from 2 to 10 signifies adsorption process is good. The Freundlich isotherm model can be expressed according to the following equations:

7 $$\mathrm{q}\text{e}={\text{KF}} \, {\mathrm{C}{\text{e}}}^{1/n},$$

8 $$\mathrm{log}\,\mathrm{q}{\text{e}}=\mathrm{log}\,\mathrm{K}{\text{F}}+\frac{1}{\mathrm{n}}\mathrm{log\,C}{\text{e}},$$

where, q_e_ is the amount of CR adsorbed onto composite beads at equilibrium (mg/g); Ce is the final concentration of CR dyes at equilibrium (mg/L); kF and n are the Freundlich constant for adsorption capacity and adsorption intensity, respectively.

For more precise results for adsorption energy, Dubinin–Radushkevich (D–R) isotherm (Supplementary Fig. S1) was applied to energetically evaluate the adsorption type^58^. Whereas, the adsorption process can be represented as chemical adsorption if the adsorption energy (E) is higher than 8 kJ mol^−1^, while the physical adsorption takes place at E lesser than 8 kJ mol^−1^ as represented by the following equation:

9 $$\mathrm{Ln}\,{\mathrm{q}}_{\mathrm{e}}=\mathrm{Ln }\,{\mathrm{q}}_{\mathrm{s}}-{\mathrm{K}}_{\mathrm{ad }}\,{\upvarepsilon }^{2},$$

where, q_s_ is the saturation capacity, K_ad_ is a constant related to the mean free energy of adsorption per mole of the adsorbate (mol^2^/KJ^2^) and ɛ is the Polanyi potential which can be obtained according to the following equation:

10 $$\upvarepsilon =RT\,ln \left(1+\left(\frac{1}{\mathrm{C}{\text{e}}}\right)\right),$$

where, T is the temperature of solution (K), R is the gases constant (8.3144 J K^−1^ mol ^- 1^) and C_e_ is concentration of CR after adsorption process.

It can be seen that the value of R^2^ in case of Freundlich (0.999) more than Langmuir (0.958) indicating that the adsorption process were accomplished by the formation of multilayer heterogeneous of CR dye onto the surface of K@AM-CTS composite beads, in addition to the adsorption process is moderate due to value of heterogeneity factor (n) is much greater than 1, but less than 2. The calculated maximum Langmuir adsorption capacity (qm) was 104.16 mg/g at room temperature. Besides, dimensionless constant (R_L_) values (0.914–0.269) for the adsorption of CR on the surface of K@AM-CTS composite beads was less than 1 and greater than zero, indicating the favorable adsorption process^59^. On the other hand, the D–R model referred that the adsorption process can be expressed by the physical adsorption process via weak Van der Waals interactions, since the calculated bonding energy ($\mathrm{E}=\frac{1}{\sqrt{2{\mathrm{K}}_{\mathrm{ad}}}})$ < 8 kJ/mol.

### Adsorption kinetics

The study of adsorption kinetics was carried out to determine the correlation between CR adsorbed onto K@AM-CTS composite beads. Data attained from the adsorption experiments were analyzed by three well-known models; the pseudo-first order, the pseudo-second order and intra-particle diffusion. The pseudo-first order (Eq. 11), describes the kinetics of liquid–solid phase adsorption, in which the rate of adsorption occupied positions proportional to number of active site of K@AM-CTS composite beads^60^. The pseudo-second order model is essential to determine theoretical adsorption capacity at equilibrium time, which can be represented by (Eq. 12)^61^. Furthermore, Intra-particles diffusion model (Eq. 13) displays the adsorption mechanism which could involves a multi-step for transport the molecules of CR dye from the aqueous phase to the surface of K@AM-CTS composite beads. Consequently, the diffusion of CR dye molecules into the interior of the pores of composite beads took place^62^. Besides, the diffusion coefficient (C) of CR dye in the bulk side could be valuated a good impression about the hurdle layer thick, which is significant when C value higher than zero^63^.

11 $$\mathrm{ln}\left(\mathrm{q}{\text{e}}-\mathrm{q}{\text{t}}\right)=\mathrm{ln}\,\mathrm{q}{\text{e}}-\mathrm{K}_{1}\mathrm{t},$$

12 $$\frac{t}{\mathrm{q}{\text{t}}}=\frac{1}{\mathrm{K}{2}{\mathrm{q}{\text{e}}}^{2}}+\left(\frac{1}{\mathrm{q}{\text{e}}}\right) t,$$

13 $$\mathrm{q}{\text{t}}=\mathrm{k}{\text{p}}{\mathrm{t}}^{1/2}+C,$$

where, q_e_ and q_t_ are the uptake of CR by K@AM-CTS at equilibrium and time respectively (mg/g). K_1_ is the rate constant of pseudo-first order (min^−1^), K_2_ is the rate constant of the pseudo-second order (g mg^−1^ min^−1^). Kp represents the constant of intra-particle diffusion (mg/g min^−1/2^), and C refers to the intercept correlated to the adsorption steps suggesting boundary layer (mg g^−1^).

**Figure 9:**
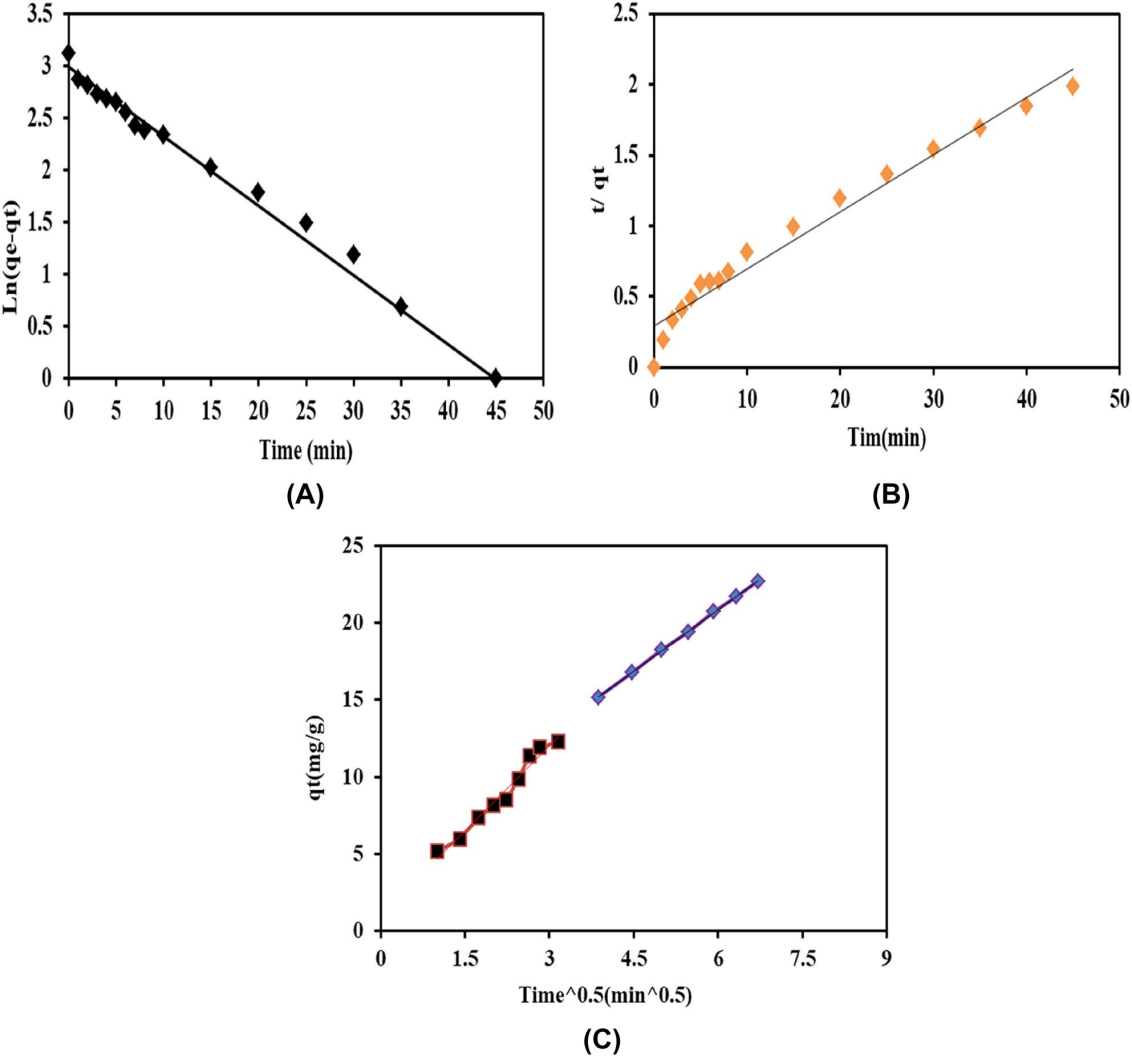
Adsorption kinetics of CR dye onto K@AM-CTS composite beads; (**A**) the pseudo-first order kinetic model, (**B**) the pseudo-second order kinetic and (**C**) Intra-particles diffusion model.

Figure 9 displayed the gained results of the pseudo-first order, the pseudo-second order and intra-particle diffusion, while the kinetics parameters were summarized in Table 3. The results clarified that the pseudo-first order (Fig. 9A) is the best fitting model for adsorption of CR dye compared to the pseudo-second order (Fig. 9B). The theoretical value of adsorption capacity (q_e,cal_) that obtained from the pseudo-first order was more closer to the experimental of adsorption capacity at equilibrium time (q_e,exp_). Furthermore, the adsorption process is fast on outer surface of composite beads at the first 10 min, followed with steady the adsorption, since the intra-particle distribution is the rate-limiting step (Fig. 9C). Lastly, the diffusion process delays the equilibrium stage as a result of decreasing the adsorption concentration in bulk. The plot of q_t_ vs t_0.5_ showed that the straight lines did not pass by the origin, while the positive intercept value (C) provides suggestion about the established boundary layer^59^. Besides, the intercept value (C) in the second stage is higher than first stage. These observations explained verified that diffusion of CR dye molecules into the outside layer of composite beads is faster than intra-particle diffusion, proving the intra-particle diffusion slow step^64^.

**Table 3:** The parameters of kinetics models for adsorption of CR dye onto K@AM-CTS composite beads.

Kinetics model	Parameter	Value	
Pseudo-first order	q_e,cal_ (mg/g)	19.80	
	k_1_ (min^−1^)	0.0667	
	R^2^	0.9849	
Pseudo-second order	q_e,cal_ (mg/g)	24.81	
	k_2_ (g mg^−1^ min^−1^)	0.00551	
	R^2^	0.9683	
Intra-particles diffusion		First step	Second step
	K_p_ (mg g^−1^ min^−1/2^)	3.642	2.656
	C (mg g^−1^)	1.0786	4.9127
	R^2^	0.97	0.999

### Adsorption thermodynamics studies

The thermodynamic study is very important to clarify the influence of temperature on the adsorption performance as well as to explore the nature of adsorbents. The parameter of thermodynamics include the standard free energy change (ΔG°), entropy change (ΔS°) and change in enthalpy (ΔH°) as presented in Eqs. (14–17) ^65^. Values of ΔH° and ΔS° are calculated from the slope and intercept of the line plotted by ln Kc against 1/T as shown in Supplementary Fig. S2.

14 $${\mathrm{K}}_{\mathrm{c}}=\frac{\mathrm{q}{\text{e}}}{\mathrm{C}{\text{e}}},$$

15 $${\mathrm{ln\,K}}_{\mathrm{c}}=\frac{\Delta {\mathrm{S}}^{\mathrm{o}}}{\mathrm{R}}-\frac{\Delta {\mathrm{H}}^{\mathrm{o}}}{\mathrm{RT}},$$

16 $${\Delta \mathrm{G}}^{\mathrm{o}}={\Delta \mathrm{H}}^{\mathrm{o}}-\mathrm{T}{\Delta \mathrm{S}}^{\mathrm{o}},$$

17 $${\Delta \mathrm{G}}^{\mathrm{o}}=-RT\,{\mathrm{ln\,K}}_{\mathrm{c}},$$

where, Kc is the thermodynamic equilibrium constant, q_e_ and C_e_ are the CR concentration on the K@AM-CTS at equilibrium (mg/g) and concentration of CR remained in the solution after adsorption process at equilibrium (mg/g) respectively. R is the gas constant (8.314 J mol^−1^ K^−1^) and T is the temperature of adsorption (K).

As depicted in Table 4, the calculated parameters from Van't Hoff equation (Eq. 13), both of ∆H^o^ and ∆S^o^ displayed positive values, confirming that the adsorption of CR onto composite beads is endothermic in nature and randomness process. In addition, the value of ∆H^o^ is less than 40 kJ/mol, suggesting the physical adsorption process^31^. Meanwhile, the adsorption of CR dye is spontaneous due to the negative values of ΔG° at different temperatures^28^, since it changed from − 5.655 to − 8.463 kJ mol^−1^ by increasing temperature from 298 to 328 K.

**Table 4:** Thermodynamic coefficients for adsorption CR onto K@AM-CTS composite beads.

Temperature (K)	ΔG° (kJ/mol)	ΔH° (kJ/mol)	ΔS° (J/mol K)
298	− 5.655	22.080	0.093
308	− 6.223		
318	− 7.146		
328	− 8.463		

### Comparison with other adsorbents

Table 5 represents a comparison between the created K@AM-CTS composite beads and other adsorbents for removal of CR dye. It was noticeable that Kaolin@AM-CTS composite beads achieved the best adsorption capacity (104.16 mg/g) at equilibrium time (45 min). The higher adsorption capacity could ab a result of the more electrostatic interactions between positive extra amine groups on surface of composite beads and the anionic groups of CR dye compared to the other presented adsorbents.

**Table. 5.** Comparison of various adsorbents for adsorption of CR dye.

Adsorbent	q_max_ (mg/g)	pH	Equilibrium time (min)	References
Cabbage waste powder	2.31	8	180	^66^
Chitosan–vanadium-titanium-magnetite composite	90.91	6	720	^4^
Kaolin	5.44	7.5	1440	^47^
Coal-based mesoporous activated carbon	53	10	1800	^15^
Modified commercial zeolite catalyst	21.11	7	90	^67^
Bentonite and Modified Bentonite	25.38	–	60	^14^
Chitosan hydro-beads	93	6	1440	^68^
Magadiite-chitosan composite beads	200	6.5	210	^69^
Bagasse fly ash and activated carbon	11.88	7	240	^70^
Acid activated red mud	7.08	7	90	^71^
K@AM-CTS composite beads	104.16	7	45	This work

### The proposed adsorption mechanism

**Figure 10:**
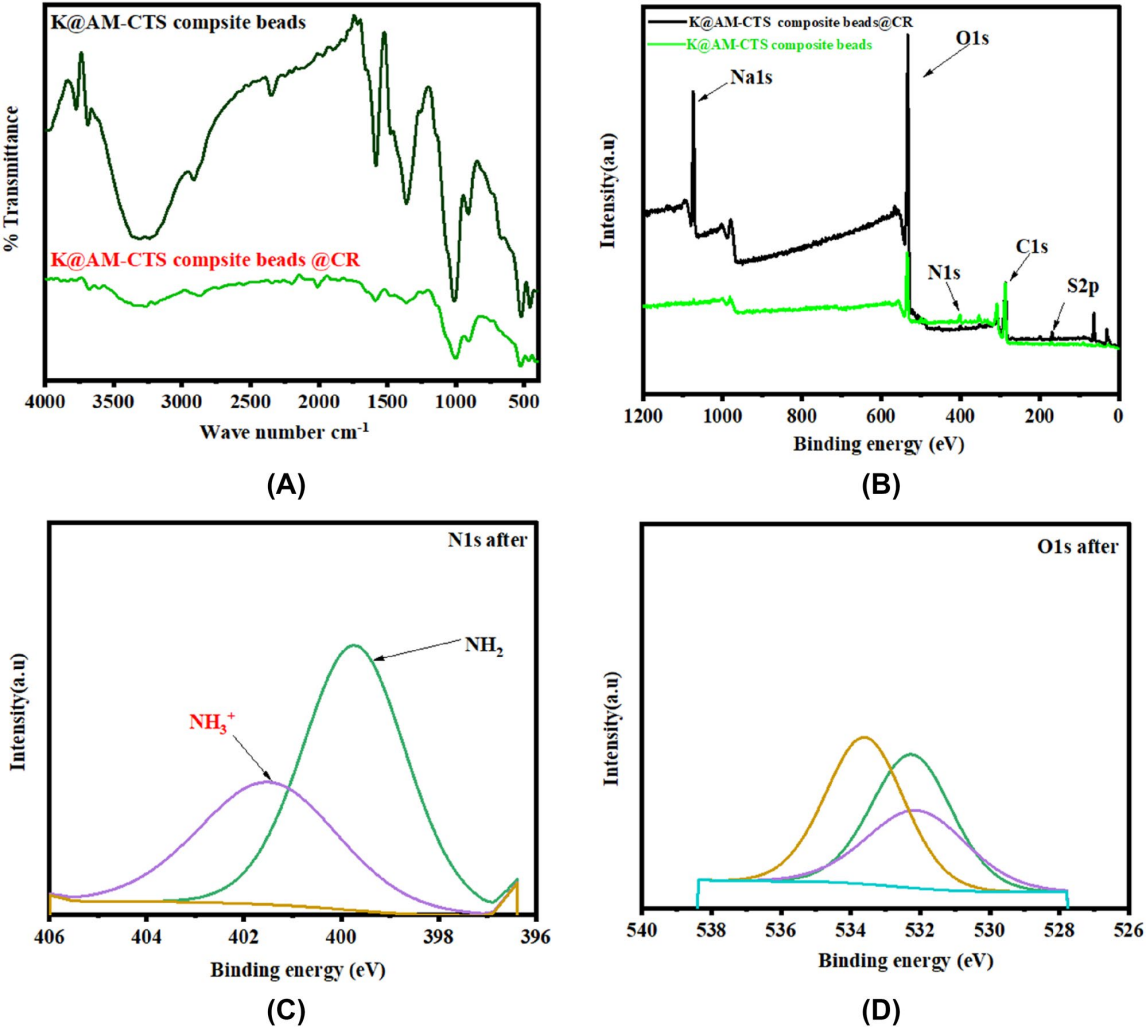
(**A**) FTIR of K@AM-CTS composite beads before and after adsorption, (**B**) XPS spectra of K@AM-CTS composite beads before and after adsorption, (**C**) N1s and (**D**) O1s after adsorption of CR dye.

The mechanism of adsorption CR dye onto K@AM-CTS composite beads adsorbent was concluded according to the gained results of FTIR, XPS and SEM analysis after the adsorption process. FTIR spectrum of K@AM-CTS composite bead after adsorption of CR dye (Fig. 10A) demonstrate the unique peaks at band 1588 and 1475 cm^−1^, which assigned to C=C and N=N stretching of the benzene ring of CR dye molecule^72^. The detected band at 1361 cm^−1^ could be ascribed to the C–N bending^69^, while the new band at 1243 cm^−1^ is assigned to S=O stretching vibration of sulfonic acid. Besides, the observed shift in the broad bands of –NH_2_/or –OH^−^ groups from 3312 to 3338 cm^−1^ could be associated with the adsorption of CR dye onto K@AM-CTS composite beads via the electrostatic interactions with the anionic SO_3_^−^ groups of CR dye. The enhancement of the adsorption was occurred also via the formation of hydrogen bonding^73^. On the other hand, XPS analysis of K@AM-CTS composite beads before and after adsorption of CR dye was illustrated in wide-spectrum as shown in Fig. 10B. A new peaks of S2p and Na1s of CR structure were appeared after the adsorption process at BE values of 1171 and 168 eV compared to the main elements of the composite, indicating that CR dye molecules were effectively adsorbed onto K@AM-CTS composite beads.

The high-resolution spectrum of N1s after adsorption was depicted in Fig. 10C, which demonstrated a new peak at BE 402.5 eV as a result of generation of NH_3_^+^ in acid medium. Hence, the electrostatic attractions occurred between the negatively charged –SO3^−^ groups on the surface of CR dye and NH_3_^+^ on the surface of K@AM-CTS composite beads^74^. The high-resolution spectrum of O1s after the adsorption of CR dye (Fig. 10D) illustrated that the intensities of –OH^−^ and Si–O–Si peaks were decreased due to the ion exchanging of OH and Si groups with CR dye^31^.

**Figure 11:**
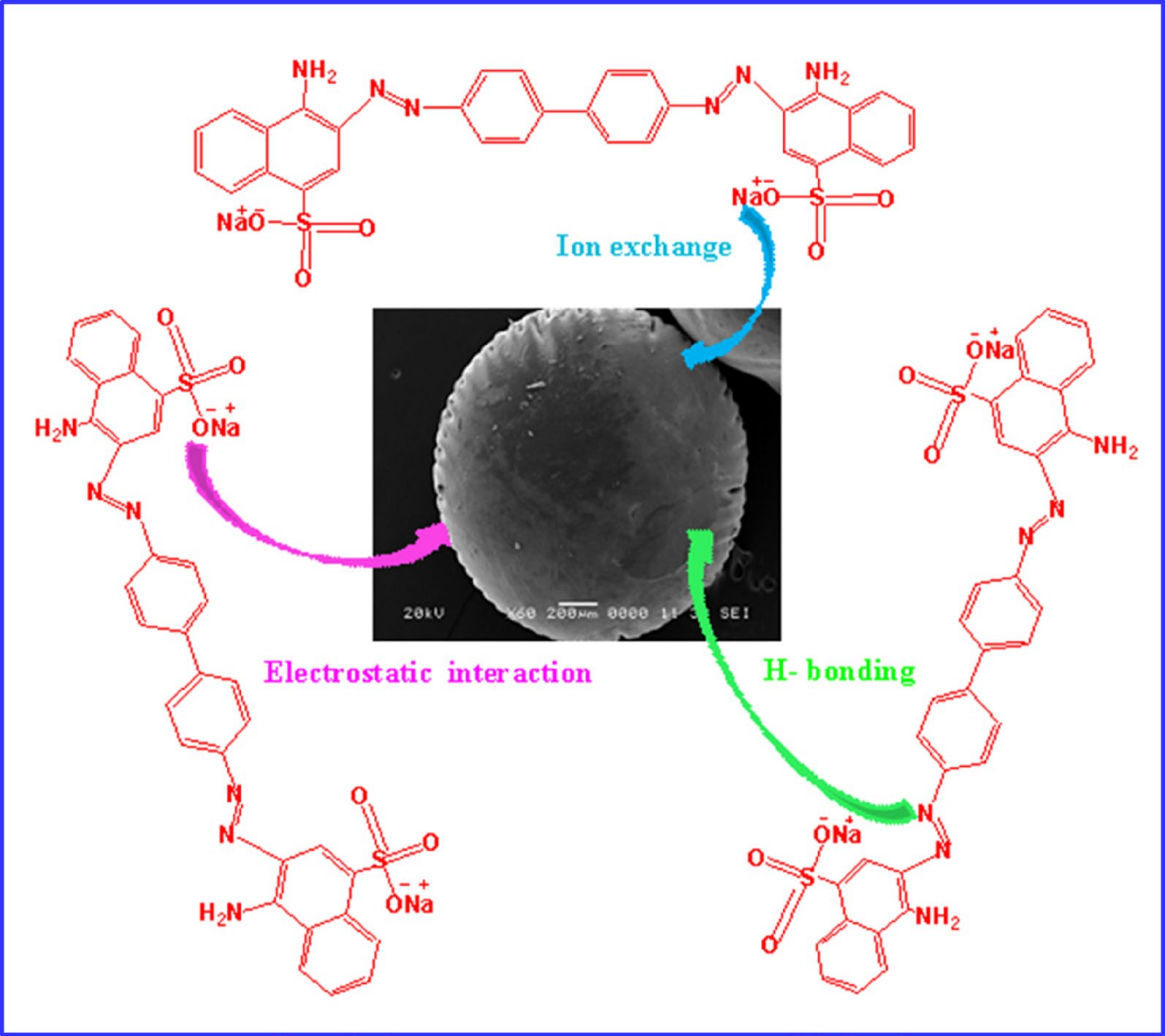
Proposed mechanism for the adsorption of CR dye onto K@AM-CTS composite beads.

Figure 11 clarified that the SEM of K@AM-CTS composite beads showed a relatively smooth surface after adsorption of CR dye compared to irregular and rougher surface of composite beads before adsorption (Fig. 4E)^48^. In addition, Fig. 11 also displayed the proposed adsorptive removal mechanism of anionic CR dye, which involves the electrostatic interactions, ion exchanging and H-bonding.

## Conclusion

This study reported the formulation of new kaolin incorporated aminated chitosan composite beads for adsorptive removal of anionic Congo red dye from aqueous solution. The characterization stage indicated that that the K@AM-CTS composite beads demonstrated better thermal stability compared to pristine chitosan, in addition to the higher surface area with a positively charged surface at pH6. All parameters affecting the adsorption process were inspected in detailed. Moreover, various kinetics, isotherms and thermodynamics models were applied to elucidate the adsorption process, while the adsorption mechanism was also hypothesized. The developed displayed several advantages including eco-friendly, cheap and easy-separable. Furthermore, K@AM-CTS composite beads showed higher adsorption performance compared to other adsorbents reported in literature, in addition to their excellent reusability for eight sequential cycles. These findings substantiate that the developed composite beads could be applied as sustainable and reusable adsorbent for removing anionic dyes from industrial wastewater.

## Supplementary Information

Supplementary Information.

## Data Availability

The data presented in this study are available on request from the corresponding author.
