# Supplementary material for: Reusable kaolin impregnated aminated chitosan composite beads for efficient removal of Congo red dye: isotherms, kinetics and thermodynamics studies
Source: Sci Rep. 2022 Jul 28;12:12972. doi: 10.1038/s41598-022-17305-w (PMC9334362; doi:10.1038/s41598-022-17305-w)
Supplement: Supplementary file 1 — Supplementary Information. [file 41598_2022_17305_MOESM1_ESM.docx]

**Supplementary file**

**Table S1**: Characteristics of Congo red dye.

| Name | Chemical structure | Chemical formula | M.wt  (g/mol) | λ (nm) |
| --- | --- | --- | --- | --- |
| Congo Red  (the sodium salt of 3,3′-([1,1′-biphenyl]-4,4′-diyl)bis(4- aminonaphthalene-1-sulfonic acid) | 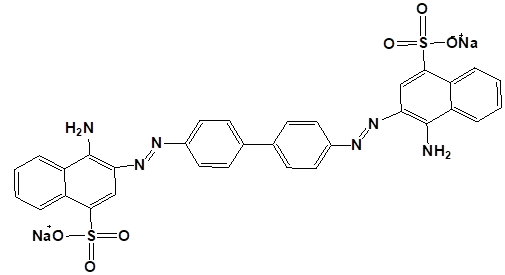 | C_32_H_22_N_6_Na_2_O_6_S_2_ | 696.6 | 497 |


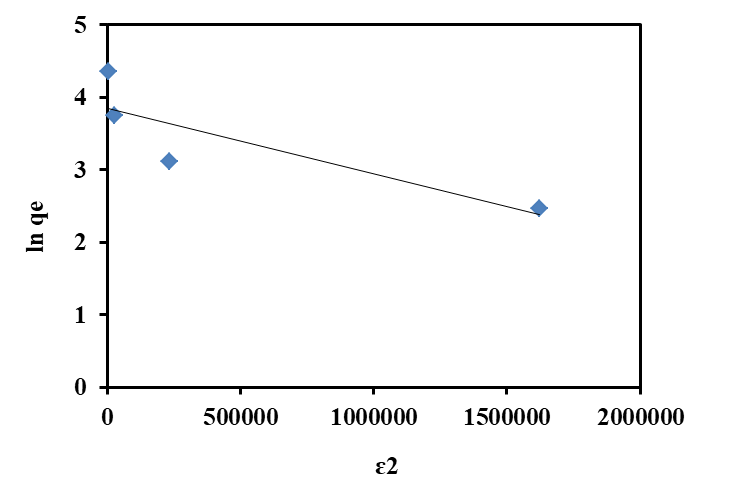


**Fig. S1.** D-R isotherm models for adsorption of CR dye on to K@AM-CTS composite beads .

**Fig. S2.** Study of adsorption process of CR on K@AM-CTS at different temperatures by Van't Hoff equation.
